# Validation of the Hungarian version of the King’s Health Questionnaire (KHQ) in women

**DOI:** 10.1038/s41598-026-49181-z

**Published:** 2026-04-24

**Authors:** Éva Szatmári, Alexandra Makai, Viktória Prémusz, Pongrác Ács, Márta Hock

**Affiliations:** 1 Doctoral School of Health Sciences, Faculty of Health Sciences, University of Pécs, Pécs, Hungary; 2 Faculty of Physical Education and Sport, Babeş-Bolyai University, Cluj-Napoca, Romania; 3 Institute of Physiotherapy and Sport Sciences, Faculty of Health Sciences, University of Pécs, Vörösmarty street, Pécs, 7621 Hungary; 4 Physical Activity Research Group, Szentágothai Research Centre, Pécs, Hungary; 5 National Laboratory on Human Reproduction, University of Pécs, Pécs, Hungary; 6 MTA-PTE Human Reproduction Scientific Research Group, Pécs, Hungary

**Keywords:** quality of life, urinary incontinence, questionnaire, Hungarian, translation, Urogenital diseases, Quality of life

## Abstract

King’s Health Questionnaire (KHQ) is a reliable and valid instrument which assesses the impact of urinary incontinence on quality of life. There is no validated Hungarian version of this questionnaire; therefore, the aim of this study was to develop a reliable and culturally sensitive Hungarian version of the KHQ. A validation study was conducted from May to October 2024. The KHQ was translated into Hungarian in accordance with international guidelines. Participants completed the Hungarian versions of the KHQ, International Consultation on Incontinence Modular Questionnaire-Urinary incontinence Short Form (ICIQ-UI SF) and 36-Item Short-Form Health Survey. Validity and reliability of the Hungarian KHQ was assessed using confirmatory factor analysis (structural validity), Cronbach’s alpha (internal consistency) and intraclass correlation coefficient (test–retest reliability). Convergent validity and discriminant validity were also assessed. Regarding structural validity, all fit indices were found acceptable. Cronbach’s alpha for most domains was above 0.8. Intraclass correlation coefficient values for each domain exceeded 0.817. Regarding correlation between domains of KHQ and ICIQ-SF, most domains of the KHQ were strongly correlated with the overall impact of urinary incontinence (last question of the ICIQ-SF). The Hungarian version of the KHQ is a valid and reliable instrument to evaluate the impact of urinary incontinence on quality of life among Hungarian women. Registered by the US National Institute of Health, Identifier NCT NCT05618184. Registration Date: November 8th, 2022. https://clinicaltrials.gov/study/NCT05618184?term=NCT05618184&rank=1.

## Introduction

Urinary incontinence (UI) is one of the most common lower urinary tract symptom, affecting women of all ages worldwide^1,2^. According to the definition suggested by The International Continence Society (ICS), UI is ’the complaint of involuntary loss of urine’^3^. Population based studies have shown that the prevalence of „any” UI in women ranged from 5 to 70%, with most studies showing 25–45% prevalence^4^. Between 2010 and 2050, the prevalence of UI is predicted to increase by 55% as a result of population aging and rising obesity^5^.

The most common type of UI is stress urinary incontinence (SUI), which is defined as the “involuntary loss of urine on effort or physical exertion (e.g. sporting activities), or on sneezing or coughing”^3^. The prevalence of SUI varies from 10% to 39% and it peaks in the fifth decade of life^1,3,6^. The other main types of UI are urgency urinary incontinence (UUI) and mixed urinary incontinence (MUI). UUI is defined as uncontrolled leakage of urine associated with urgency, while MUI is a combination of symptoms of both SUI and UUI^3^.

Between 2005 and 2006 and 2017–2018, the estimated prevalence of UUI has significantly increased from 40.9% to 49.5% among women aged 60 years or older^6^. Advancing age is a powerful predictor of UI^7^. More than half of women living in nursing homes are affected by UI, leading to worsened quality of life (QoL), increased morbidity and higher burden of health care costs^8–10^. The economic burden of UI is substantial. According to the results of multinational studies, the estimated annual cost-of-illness for UUI ranged from €2.9 billion EUR (five European countries in 2000, direct costs) to €7.0 billion EUR (Canada and five European countries in 2005, direct and indirect costs)^11^.

Pregnancy and childbirth are considered as important risk factors for UI^12^. Other risk factors related to UI include: hysterectomy, previous pelvic floor surgery, race, chronic cough, smoking^7^, constipation^13^, obesity, menopause, physical activity^4^ and work involving physical strain^1^.

UI has a serious negative effect on the psychophysical and social well-being of women^14^. Depression, anxiety, negative self-perception, embarrassment, deterioration in sexual life, decrease in physical activity and social isolation are often linked to UI. Work, entertainment and daily activities could also be impaired^15–17^. Despite the severe impact of UI on quality of life, a large number of women do not seek care. From fifty to seventy-five% of women with UI do not seek care^18–20^.

Evaluation of QoL has become an integral part of determining the impact of UI on the individual and evaluating the efficacy of treatments. Using patient-reported outcome measures to assess QoL represents a valuable source of information for both researchers and health care professionals^1^.

King’s Health Questionnaire (KHQ) is a self-administered, disease-specific, reliable and valid instrument which assesses the impact of UI on QoL^21^. It was developed in English and later adapted into Portugese^22^, Brazilian^23^, Turkish^24,25^, German^26^, Polish^27^, Spanish^28^, Marathi^29^, Thai^30^ and Japanese^31^. The KHQ also proved to be a valuable evaluation tool after UI surgery^32^.

There are a limited number of validated questionnaires about UI in Hungarian^33,34^. Therefore, the aim of this study was to translate the KHQ into Hungarian and to develop a valid, reliable and culturally sensitive Hungarian version of the KHQ.

## Methods

This validation study was conducted from May to October 2024. The study protocol was registered by the US National Institute of Health (clinicaltrials.gov, Identifier NCT NCT05618184) and approved by the National Scientific and Ethical Committee (lV/1596-3/2022/EKU).

### Participants

Participants were recruited from the Thermal Rehabilitation Centre Harkány, Pécs, Hungary. We also forwarded an invitation to participate in the study via e-mail to different primary care clinics. The purpose of the study and the online survey link were included in the invitation. Participation in the study was voluntary. Before completing the self-administered questionnaire, all participants provided their written informed consent. No identifying information was collected from the study participants. Inclusion criteria included Hungarian women, aged 18 years or older, having symptoms of UI and ability to read and write Hungarian, whereas exclusion criteria were urinary tract infections, neurological disorders associated with muscle weakness and malignancy. We also selected women without UI to form a control group. Controls were defined as Hungarian women aged 18 years or older, with the ability to read and write Hungarian. Women were excluded from the control group if they had symptoms of UI.

### Outcome measures

Participants completed the Hungarian versions of the KHQ, International Consultation on Incontinence Modular Questionnaire-Urinary incontinence Short Form (ICIQ-UI SF) and 36-Item Short-Form Health Survey (SF-36).

KHQ is a self-administered questionnaire which assesses the impact of UI on QoL. It is composed of 21 items divided into nine different domains: general health perception, incontinence impact, role limitations, physical limitations, social limitations, personal relationships, emotions, sleep/energy and severity measures. Each domain is scored separately ranging from 0 (best QoL) to 100 (worst QoL). The KHQ has shown good validity and reliability. Based on the opinion of women who completed it, the KHQ is easy to use, relevant and comprehensive without being bothersome^35,36^.

The ICIQ-UI SF measures the severity of UI by assessing the frequency of UI, leakage amount, overall impact of UI and type of UI. The total score can range from 0 to 21, with higher scores indicating more severe symptoms. It can be divided into four severity categories: slight (1–5), moderate (6–12), severe (13–18) and very severe (19–21)^37–39^.

The SF-36 assesses generic health-related QoL. It contains eight different domains: physical functioning, role limitations due to physical health, role limitations due to emotional problems, energy/fatigue, emotional well-being, social functioning, pain and general health. Each domain is scored separately ranging from 0 (worst QoL) to 100 (best QoL)^40,41^.

### Translation and cultural adaptation

The owners of the copyright of KHQ© are Linda Cardozo and Con Kelleher, 1997, all rights reserved. Before the translation process was conducted, the agreement from the MAPI Research Trust to perform the cultural adaptation of the KHQ into Hungarian was obtained. The validation of the questionnaire followed Beaton’s six-stage process and the COSMIN guideline: translation, synthesis, back-translation, expert committee review, pretesting, and assessment of the psychometric properties^42,43^.

#### Stage l: Initial translation 

The KHQ was independently translated into Hungarian by two Hungarian physiotherapists who were fluent in English. As recommended, the two translators had different work experience: only the first translator had worked with patients with UI. Each translator produced a written report of the translation they had completed. During the translation, efforts were made to preserve the original wording and conceptual meaning of each item as closely as possible.

#### Stage ll: Synthesis of the translation

The written reports and the two independent translations were evaluated by the research team and the two forward translators. The research team consisted of three physiotherapists with experience in working with women who have UI, and a professional translator. A synthesis of the initial translations was conducted (version 1).

#### Stage lll: Back translation

The back translation into English was performed by a native English speaker who was fluent in Hungarian and had no prior knowledge of the KHQ. The back-translator was not informed of the concepts explored.

#### Stage lV: Expert committee

The study team and the translators (forward and back translators) compared the back-translation to the original English version and agreed on a second Hungarian version (version 2). After that, all versions of the questionnaire were carefully reviewed by the study team and the translators in order to achieve semantic, idiomatic, experiential and conceptual equivalence. Contact was maintained with the owners of the original version of the KHQ throughout the process to clarify any possible concerns. All identified issues were discussed until consensus was reached.

#### Stage V: Test of pre-final version

The questionnaire was pre-tested by conducting cognitive interviews within the target population to assess the adequacy and clarity of the vocabulary used. Out of the sixty-eight women who were contacted, 20 agreed to participate. Cognitive interviews were led by the same physiotherapist with experience in working with women who have UI. Women were asked to read questions aloud and explain what they understood^44^. After making minor adjustments based on the participants’ feedback, the Hungarian version of the KHQ was finalized. Both linguistic fidelity and cultural relevance of the final Hungarian version of the questionnaire were ensured.

The Hungarian KHQ and documentation from earlier stages were sent to the MAPI Research Trust for appraisal. The Hungarian version of the KHQ can be obtained from the MAPI Research Trust (https://eprovide.mapi-trust.org) by submitting a request.

#### Stage Vl: Assessment of the psychometric properties

The psychometric evaluation of the Hungarian KHQ followed the recommendations of the Cosmin guideline^43^. Structural validity, internal consistency, test–retest reliability, convergent validity and discriminant validity were assessed in order to determine the reliability and validity of the translated questionnaire.

### Statistical analysis

Statistical analysis was performed using SPSS version 28 and SPSS AMOS version 29.0. A descriptive analysis was performed to present the data (for categorical variables absolute number and percentage, for scales mean and standard deviation). A p-value of less than 0.05 was considered statistically significant. The predetermined minimal sample size was based on a ratio of ten participants per item for confirmatory factor analysis^45^. Baseline characteristics were compared between the UI and the control group by using the Pearson’s chi square test of association and the Mann–Whitney U test.

To assess structural validity, confirmatory factor analysis was carried out. The following fit statistics were included in the analysis: χ^2^, degree of freedom (df) and χ^2^ divided by df (χ^2^/df ratio; <3.00 is adequate, < 5.00 is acceptable). Comparative fit index (CFI; >0.90 is acceptable), Tucker-Lewis index (TLI; >0.90 is acceptable), root mean square error of approximation (RMSEA; < 0.08 is considered good fit, 0.08 < RMSEA < 0.1 indicates adequate fit) and standardized root mean square residual (SRMR; ≤0.08 is acceptable) were also used^46,47^.

Internal consistency was assessed by using Cronbach’s alpha, with values above 0.70 considered good internal consistency^48^. The test–retest reliability of the questionnaire was analyzed using intraclass correlation coefficient (ICC), values between 0.5 and 0.75 considered moderate reliability, between 0.75 and 0.9 indicating good reliability and greater than 0.9 considered excellent reliability)^49^. For test–retest reliability, data were collected by face-to-face interviews with 25 women. Duration of the test–retest period was fourteen days.

Convergent validity was calculated using Spearman’s correlation analysis between the KHQ, SF-36 and ICIQ-SF. Values ranging from 0.1 to 0.3 were considered weak, 0.3 to 0.5 moderate and ≥ 0.5 strong^50^.

To assess discriminant validity, the score of the Hungarian KHQ was compared between women with and without UI using Mann–Whitney U test. We anticipated that women with UI have substantially higher KHQ scores compared to women in the control group.

## Results

Initially, a total of 281 respondents returned the questionnaire. Among them, 17 respondents did not meet the inclusion criteria and 14 women did not complete the KHQ. Overall, 250 women were included in the study. The participants were divided into two main study groups: UI group (*N* = 110) and control group (*N* = 140). The main characteristics of study participants are summarized in Table 1.

**Table 1:** Main characteristics of study participants (*N* = 250).

Characteristics	UI group (*N* = 110)	Control group (*N* = 140)	*P* value
Age, mean (SD), years	42.08 ± 17.08	27.74 ± 13.04	< 0.001
BMI, mean (SD), kg/m^2^	25.59 ± 6.04	22.30 ± 3.58	< 0.001
*Highest education level, N (%)*			
Eight grade or less	5 (4.59%)	1 (0.71%)	0.017
High school	46 (42.20%)	80 (57.14%)	
College /University	58 (53.21%)	59 (42.14%)	
Married, N (%)	50 (45.45%)	35 (25%)	< 0.001
Constipation, N (%)	30 (27.27%)	16 (11.51%)	< 0.001
Smoking, N (%)	93 (84.55%)	41 (29.50%)	0.009
Currently pregnant, N (%)	5 (4.55%)	5 (3.68%)	0.731
*Parity, N (%)*			
Nulliparous	39 (35.45%)	98 (70%)	< 0.001
Parous	71 (64.55%)	42 (30%)	
Vaginal childbirth, N (%)	61 (55.45%)	28 (20%)	< 0.001
Caesarean section, N (%)	13 (12.38%)	14 (10.69%)	0.685
Vacuum /Forceps delivery, N (%)	7 (6.36%)	2 (1.43%)	0.038
Perineal injury/rupture, N (%)	40 (38.10%)	12 (9.16%)	< 0.001
Episiotomy, N (%)	53 (50%)	28 (21.37%)	< 0.001
Birth weight greater than 4000 g, N (%)	13 (12.38%)	4 (3.05%)	0.006
*Types of urinary incontinence, N (%)*			
Stress urinary incontinence	69 (63.89%)	–	
Urge urinary incontinence	18 (16.67%)	–	
Mixed urinary incontinence	21 (19.44%)	–	

Results of the CFA revealed the following values: χ^2^ = 354.404, DF = 131, *p* < 0.001, χ^2^ /DF = 2.705, CFI = 0.923, TLI = 0.90, RMSEA = 0.107, and SRMR = 0.025.

The Cronbach’s alpha for each domain except for the domain of sleep and energy was above 0.8. The results of the test–retest analysis showed high and significant correlations. Regarding ICC, values ranged from 0.817 (sleep and energy domain) to 0.945 (emotions domain). The Cronbach’s alpha values and the ICC for all domains of the Hungarian KHQ are shown in Table 2.

**Table 2:** Internal consistency and test–retest reliability of the Hungarian KHQ.

KHQ	Cronbach’s alpha	ICC	95% CI
General health perception	–	0.906	0.794–0.958
Incontinence impact	–	0.886	0.760–0.948
Role limitations	0.894	0.934	0.834–0.972
Physical limitations	0.845	0.889	0.754–0.950
Social limitations	0.913	0.920	0.828–0.964
Personal relationships	0.928	0.872	0.733–0.941
Emotions	0.933	0.945	0.880–0.976
Sleep and energy	0.551	0.817	0.631–0.915
Severity	0.842	0.874	0.735–0.942

Regarding correlation between domains of KHQ and SF-36, there was a strong correlation between the domains of general health perception (KHQ) and general health (SF-36). All domains of KHQ except for the domain of sleep and energy were strongly correlated (r_s_ ≥ 0.5) with the domain of physical functioning (SF-36). Between the other domains, there were weak (r_s_ < 0.3) and moderate correlations (0.3 ≤ r_s_ < 0.5). Spearman’s correlation coefficients between the KHQ and the SF-36 with related p-values are shown in Table 3.

**Table 3:** Correlations between the Hungarian KHQ and the SF-36 (Spearman’s rho).

KHQ domains	SF-36 domains										
	General health	Physical functioning	Role limitations due to physical health	Social functioning	Emotional well-being	Pain	*P* value	Energy or fatigue	*P* value	Role limitations due to emotional problems	*P* value
General health perception	− 0.634	− 0.504	− 0.357	− 0.421	− 0.425	− 0.364	< 0.01	− 0.398**	< 0.01	− 0.219	< 0.01
Incontinence impact	− 0.363	− 0.591	− 0.298	− 0.222	− 0.207	− 0.163	< 0.01	− 0.105	> 0.05	− 0.094	> 0.05
Role limitations	− 0.356	− 0.598	− 0.308	− 0.262	− 0.210	− 0.180	< 0.01	− 0.163*	< 0.05	− 0.123	> 0.05
Physical limitations	− 0.312	− 0.566	− 0.365	− 0.249	− 0.264	− 0.252	< 0.01	− 0.234**	< 0.01	− 0.175	< 0.01
Social limitations	− 0.452	− 0.607	− 0.358	− 0.248	− 0.285	− 0.214	< 0.01	− 0.196**	< 0.01	− 0.154	< 0.05
Personal relationships	− 0.409	− 0.551	− 0.325	− 0.243	− 0.277	− 0.251	< 0.01	− 0.223**	< 0.01	− 0.126	> 0.05
Emotions	− 0.428	− 0.569	− 0.393	− 0.277	− 0.262	− 0.207	< 0.01	− 0.209**	< 0.01	− 0.167	< 0.05
Sleep and Energy Disturbances	− 0.325	− 0.376	− 0.350	− 0.334	− 0.266	− 0.226	< 0.01	− 0.294**	< 0.01	− 0.199	< 0.01
Severity measures	− 0.345	− 0.509	− 0.315	− 0.202	− 0.293	− 0.214	< 0.01	− 0.174*	< 0.05	− 0.145	< 0.05

Regarding correlation between domains of KHQ and particular measuring scales of the ICIQ-SF, all domains of KHQ except for general health perceptions were strongly correlated (r_s_ ≥ 0.5) with overall impact of UI (ICIQ-SF). There were also strong (r_s_ ≥ 0.5) and moderate correlations (0.3 ≤ r_s_ < 0.5) between other domains. All correlations were significant at *p* ≤ 0.01. Spearman’s correlation coefficients between the KHQ and the ICIQ-SF with related p values are shown in Table 4.

**Table 4:** Correlations between the Hungarian KHQ and the ICIQ-SF (Spearman’s rho).

KHQ domains	ICIQ-SF			
	Frequency of UI	Amount of leakage	Overall impact of UI	*P* value
General health perception	0.372	0.351	0.392	< 0.01
Incontinence impact	0.692	0.662	0.769	< 0.01
Role limitations	0.557	0.538	0.610	< 0.01
Physical limitations	0.603	0.604	0.687	< 0.01
Social limitations	0.538	0.493	0.643	< 0.01
Personal relationships	0.506	0.490	0.569	< 0.01
Emotions	0.650	0.569	0.679	< 0.01
Sleep and Energy Disturbances	0.492	0.471	0.547	< 0.01
Severity measures	0.643	0.604	0.713	< 0.01

Regarding discriminant validity, KHQ scores for each domain were significantly higher in the UI group (*p* < 0.001). Table 5 presents the mean score of each KHQ domain for the UI and control group.

**Table 5:** Mean score of the Hungarian KHQ domains for the UI and control group.

KHQ domains	Mean ± SD for UI group (*N* = 110)	Mean ± SD for Control group (*N* = 140)	*P* value
General health perception	41.59 ± 22.27	25.36 ± 19.66	< 0.01
Incontinence impact	31.21 ± 32.01	1.19 ± 7.38	< 0.01
Role limitations	21.96 ± 28.18	1.58 ± 6.47	< 0.01
Physical limitations	29.59 ± 31.08	4.11 ± 13.44	< 0.01
Social limitations	17.90 ± 27.03	1.21 ± 6.66	< 0.01
Personal relationships	22.03 ± 33.38	1.54 ± 7.63	< 0.01
Emotions	28.05 ± 31.87	2.81 ± 7.64	< 0.01
Sleep and energy disturbances	24.76 ± 21.07	7.37 ± 13.31	< 0.01
Severity measures	29.67 ± 23.11	6.95 ± 11.37	< 0.01

*UI group* urinary incontinence group.

## Discussion

KHQ is a self-administered, disease-specific, valid instrument which assesses the impact of UI on QoL in different domains^21^. This is the first study that aimed to develop a culturally sensitive, reliable and valid Hungarian version of the KHQ. According to our results, the Hungarian KHQ demonstrated adequate reliability and validity in Hungarian women. The Hungarian KHQ is the first validated tool that comprehensively and specifically assesses QoL among Hungarian-speaking women with UI.

Brusaca et al.^23^ assessed the factor structure and the structural validity of the Brazilian KHQ by using exploratory and confirmatory factor analysis. Based on their results, they found that the Brazilian KHQ had a five-factor structure (limitations of daily life, personal relationship, emotions, sleep/energy and severity measures). They also excluded item 4d and 8b since they did not meet the retention criteria. This five-factor structure explained 74% of the total variance of the questionnaire. In the study of Kaya et al.^25^, the Turkish KHQ had a three-factor structure, which explained 64% of the total variance of the questionnaire. In the study of Viana et al.^35^, the Portuguese KHQ also had a three-factor structure, which explained 68% of the total variance of the questionnaire.

Regarding confirmatory factor analysis, the RMSEA value in the current study was slightly higher than the commonly excepted thresholds (RMSEA = 0.107). However, it was recommended that model fit indices should be interpreted collectively^46^. All the other fit indices in the present study were found to be acceptable (CFI = 0.923, TLI = 0.900, SRMR = 0.025).

The reliability of the Hungarian KHQ was good in terms of test–retest reliability and internal consistency. We found that ICC values for each domain exceeded 0.817. Karapolat et al.^24^ also had similar results: ICC for most subscales of the Turkish KHQ exceeded 0.84.

We found that the Cronbach’s alpha for each domain except for the domain of sleep and energy was above 0.8. In the study of Nunes Tamanini et al.^22^, Joshi et al.^29^ and Romero-Cullerés et al.^28^, the Cronbach’s alpha for the sleep and energy domain was < 0.7 (0.49, 0.49, < 0.7; respectively). In the original version, it was 0.78^21^. In other studies, it ranged from 0.74 to 0.90^24,25,27,51^. In the study of Brusaca et al.^23^ (Brazilian KHQ), the Cronbach’s alpha for the sleep and energy domain was 0.80. However, one question of the domain was modified in their study: “Do you feel worn out/tired?” (original version) was changed to “Does your bladder problem make you feel worn out and tired?”. Karapolat et al.^24^ (Turkish KHQ) also modified that question in the same way. In their study, the Cronbach’s alpha for the sleep and energy domain was 0.90. During the translation process of the Hungarian KHQ, we aimed to follow strictly the structure of the original version without any modifications. However, taking into consideration the above-mentioned results, further research is needed to clarify which form of the mentioned question is the most adequate.

The main strength of the current study is the strict adherence to established cross-cultural adaptation guidelines (Beaton’s six-stage process and the COSMIN guideline) and rigorous psychometric evaluations including structural validity, internal consistency, test–retest reliability, convergent validity and discriminant validity.

The present study had some limitations. While hypotheses regarding expected differences between clinically distinct groups were formulated in line with COSMIN recommendations, it should be noted that the groups also differed in several demographic and clinical characteristics. Moreover, we collected data from a convenience sample, leading to difficulties in generalization.

## Conclusion

In conclusion, the Hungarian version of the KHQ is a valid and reliable instrument to evaluate the impact of UI on QoL among Hungarian women. This questionnaire can be used in both clinical and research practice to assess health related QoL among Hungarian women with UI. However, separate validation is required before applying the Hungarian KHQ to male populations.

## Data Availability

The datasets used and/or analyzed during the current study are available from the corresponding author on reasonable request.
